# Dr. James Adair Lawrie

**Published:** 1860-03

**Authors:** 


DR. JAMES ADAIR LAWRIE.—The following notice is taken from the *London Lancet* of December 3, 1859:—

"The death of this distinguished surgeon will be felt as a great calamity in the sister city, and in the west of Scotland generally. Dr. Lawrie had long enjoyed a large and lucrative practice, and very many of his patients had become old personal friends. His especial professional eminence was as a surgeon, but it was his knowledge of the profession in all its departments that made him so singularly useful and successful. He was scarcely less trusted and sought after as a physician than as a surgeon. His constant kindness to the junior and poorer members of the profession will make his memory cherished by many of the race that follows him. His enlightened views on the subject of medical education led to his election, by the Universities of Glasgow and St. Andrews, as their representative in the new Medical Council."

